# Degree of Differentiation of Esophageal Squamous Cell Carcinoma and Micrometastasis to Lymph Nodes

**DOI:** 10.14789/jmj.JMJ21-0041-OA

**Published:** 2022-08-01

**Authors:** TAKANOBU ASAKURA, TAKASHI HASHIMOTO, TAKAO ANDO, NATSUMI TOMITA, TOSHIHARU MATSUMOTO, ATSUSHI ARAKAWA, MASAHIKO TSURUMARU, YOSHIAKI KAJIYAMA

**Affiliations:** 1 Department of Gastrointestinal Surgery, Juntendo University Medical School, Tokyo, Japan; 1 Department of Gastrointestinal Surgery, Juntendo University Medical School, Tokyo, Japan; 2 Department of Physiology, Juntendo University Medical School, Tokyo, Japan; 2 Department of Physiology, Juntendo University Medical School, Tokyo, Japan

**Keywords:** outcomes, differentiation, esophageal squamous cell carcinoma, micrometastasis, lymph nodes

## Abstract

**Objectives:**

The goal of the study was to examine the relationships among micrometastasis, pathological degree of differentiation and survival in patients with esophageal squamous cell carcinoma (SCC).

**Design:**

A single-center retrospective study of patients diagnosed with thoracic esophageal SCC.

**Methods:**

Immunostaining using CK13 was carried out for all lymph nodes resected by radical esophagectomy with three-field lymphadenectomy. The relationships among micrometastasis to lymph nodes, degree of differentiation and survival were investigated.

**Results:**

The 25 patients included 14 (56.0%) well-differentiated and 11 (44.0%) moderately differentiated cases. In multivariate analysis, well-differentiated cases were not related to micrometastasis (odds ratio (OR): 1.5, confidence interval (CI): 0.2-12, p=0.7). In multivariate analysis of survival, cases in pStage III or higher were likely to have shorter survival (hazard ratio (HR): 2.8, CI: 0.7-12, p=0.16), and those with micrometastasis also tended to have shorter survival (HR: 2.7, CI: 0.8-9, p=0.11)); however, well-differentiated cases were not significantly related to survival (HR: 1.5, CI: 0.4-5.5, p=0.5).

**Conclusion:**

Micrometastasis to lymph nodes may be a prognostic factor even in advanced esophageal cancer. The degree of differentiation was not related to micrometastasis or survival.

## Introduction

Esophageal squamous cell carcinoma (SCC) has a poor prognosis, with recurrence in lymph nodes and distal metastasis found frequently within a few years after surgical resection^1)^. Some of these findings may be caused by micrometastases that cannot be detected by hematoxylin-eosin (HE) staining^2)^; that is, pathological, rather than clinical, metastasis. Many studies have examined micrometastases in esophageal cancer, particularly to lymph nodes, but these studies have mostly been limited to earlier stage cancer^3-6)^. Thus, micrometastasis to lymph nodes in advanced esophageal cancer is poorly understood.

The relationship between outcomes of esophageal cancer and pathological degree of differentiation has been widely investigated. In general, well-differentiated carcinoma has a better prognosis and poorly differentiated cases have a poor prognosis^7)^. However, the relationship between micrometastasis to lymph nodes and the histopathological degree of differentiation is unknown. One study showed more micrometastases in well differentiated esophageal carcinoma^5)^, but another found opposite results^8)^, and currently, no conclusion has been reached. Therefore, we examined this relationship and that of micrometastasis to lymph nodes with prognosis in patients with esophageal SCC, including cases of advanced cancer.

## Methods

This study was approved by the clinical research committee of Juntendo University Hospital (E21- 0056-H01). Informed consent was not required because of the retrospective study design. Twenty-five patients with esophageal cancer underwent esophagectomy in our department at Juntendo University Medical School from January to December 2000. The inclusion criteria were [1] SCC, [2] no preoperative chemotherapy, [3] three-field lymphadenectomy, [4] completion of follow-up, and [5] complete R0 resection. The exclusion criteria were >10 metastases to lymph nodes detected by conventional HE stain.

### Surgery

All patients underwent esophagectomy with three-field lymphadenectomy by right thoracotomy and laparotomy, as reported previously9). All patients also underwent lymphadenectomy along the bilateral recurrent laryngeal nerves and around the supraclavicular area. The gastric tube was pulled up through the retrosternal route and a hand-sewn esophagogastrostomy was created in the neck.

### Postoperative adjuvant therapy

Patients who were pathologically confirmed to have ≥3 lymph node metastases received two courses of postoperative adjuvant therapy with docetaxel, cisplatin and 5-fluorouracil^10)^.

### Immunostaining

For histopathological examination of resected lymph nodes, samples were subjected to formalin fixation and paraffin embedding. Five neighboring tissue sections (3 μm) were prepared from each slice and 3 central sections were used for staining. After deparaffinizing each section, antigen retrieval was carried out by autoclave treatment (1.2 atm, 10 min). Immunostaining was performed using anti-cytokeratin13 Ks 13.1 mouse monoclonal antibody (CK13, American Research Products) diluted 20 times with antibody diluent (1% BSA/PBS) using automated staining equipment (Ventana NX System, Ventana). After immunostaining, background staining was performed with Mayer’s hematoxylin for microscopic examination. Of the cells within the lymph node capsules, nucleated cells with evenly cytokeratin-stained cytoplasm were defined as metastasis-positive.

### Clinicopathologic parameters

Data for clinicopathologic parameters, including tumor stage by TNM staging (ver. 8)^11)^, were obtained retrospectively from a hospital database. TNM staging reflects the result of HE stain. The degree of differentiation was evaluated using most parts of the tumor.

### Statistical analysis

Comparisons of two groups were performed by chi-square test. Survival curves were estimated with the Kaplan-Meier method and significant differences in survival rate were analyzed by log-rank test. Multivariate analysis was performed using logistic regression analysis for categorical data and Cox regression analysis for survival. Significance was defined as P<0.05 in all analyses. Due to the small number of cases, a result with P<0.2 was considered not to be significant, but to show a tendency. All calculations were performed using IBM SPSS Statistics ver. 23.0.

## Results

The characteristics of the 25 patients in the study are shown in Table 1. The most common disease was middle thoracic esophageal cancer, pT3, pN1, pStage III. There were 14 (56%) well-differentiated, 11 (44%) moderately differentiated, and no poorly differentiated cases. Intramural metastasis was found in resected specimens in 4 patients.

**Table 1:** Patient background

Variable		Number of patients	Micrometastasis-positive	Micrometastasis-negative	P
Sex	Male/Female	23/2	8/2	15/0	0.15
Site	Ut/Mt, Lt	3/22	1/9	2/13	0.654
Age	<60 / ≥60	12/13	6/4	6/9	0.284
Depth	pT1/pT2-pT3	11/14	2/8	9/6	0.048
Lymph node metastasis	pN0-N1/pN2-N3	14/11	2/8	12/3	0.003
Advanced stage	pStage0-Ⅱ/pStageⅢ-Ⅳ	11/14	2/8	9/6	0.048
Differentiation	well/mod	14/11	8/2	6/9	0.048
Lymphovascular invasion	ly0-2/ly3	20/5	5/5	15/0	0.002
Venous invasion	v0-1/v2	15/10	3/7	12/3	0.012
Intramural metastasis	IM0/IM1	21/4	6/4	15/0	0.008

Ut: upper thoracic esophagus, Mt: middle thoracic esophagus, Lt: lower thoracic esophagus, T: tumor, N: lymph node, well: well-differentiated squamous cell carcinoma, mod: moderately differentiated squamous cell carcinoma, ly: lymphovascular invasion, v: venous invasion, IM: intramural metastasis

### Conventional lymph node metastases

A total of 2,915 lymph nodes were collected from the 25 patients. The median number of lymph nodes per patient was 116. In histopathologic examination using HE stain, metastases were detected in 70 lymph nodes in 17 patients.

### Micrometastasis to lymph nodes

In immunostaining with CK13, one micrometastasis was detected in one patient without evidence of lymph node metastasis on HE stain, and 16 micrometastases were detected in 9 patients who were determined to be metastasis-positive by HE stain. Therefore, 10 patients were evaluated as lymph node micrometastasis-positive. The main tumors stained positive for CK13 in all 25 cases. All lymph nodes that were found to be metastasized by HE stain also stained positive with CK13.

### Relationships between micrometastasis and pathological factors

The micrometastasis-positive group included many cases with high pT and pN, advanced pStage, lymphatic and vascular invasion, and well-differentiated carcinoma (Table 1). A comparison based on differentiated types showed that well-differentiated cases were likely to have more advanced tumors (Table 2). To investigate the factors related to micrometastasis, logistic regression analysis was performed with pStage and degree of differentiation as independent variables and micrometastasis as the dependent variable. This analysis showed a tendency for cases in pStage III or higher to be more likely to be micrometastasis-positive (odds ratio (OR): 6.6, confidence interval (CI): 0.8-54, p=0.08). The OR for well-differentiated tumors was 1.5 (CI: 0.2-12), but the p value of 0.71 indicated that the relationship between degree of differentiation and micrometastasis was not significant (Table 3).

**Table 2:** Histopathologic factors and degree of differentiation

	Well differentiated	Moderately differentiated	p
T1/T2-3	2/12	9/2	0.001
N1/N2-3	3/11	7/4	0.032
Micrometastasis(+)/micrometastasis(-)	8/6	2/9	0.048
pIM0/pIM1	10/4	11/0	0.053
ly0-2/ly3	10/4	10/1	0.085
v0-1/v2	5/9	10/1	0.005

T: tumor, N: lymph node, IM: intramural metastasis, ly: lymphovascular invasion, v: venous invasion

**Table 3:** Logistic regression analysis of relationships between micrometastasis and pathological factors (degree of differentiation and Stage)

		Odds ratio	Confident intervals	p
Degree of differentiation	Well vs. moderate	1.5	0.2 - 11.5	0.71
Stage	III, IV vs. I, II	6.6	0.8 - 55	0.079

### Long-term survival

**Figure 1:**
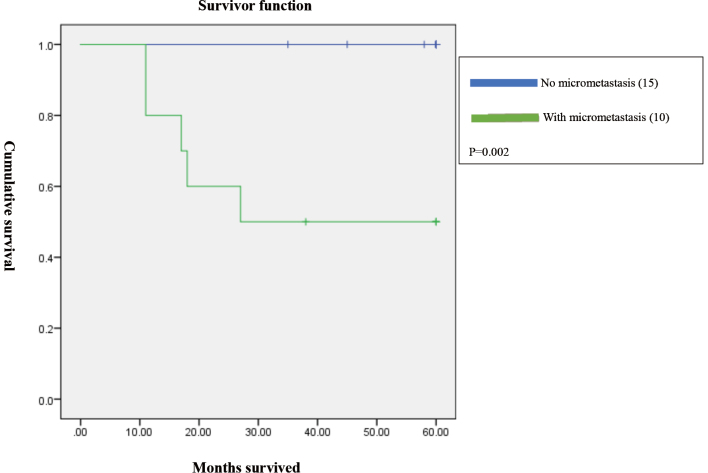
Survival curves for patients with and without micrometastasis. There was a significant difference in survival rate between those with and without micrometastasis (p=0.002).

**Figure 2:**
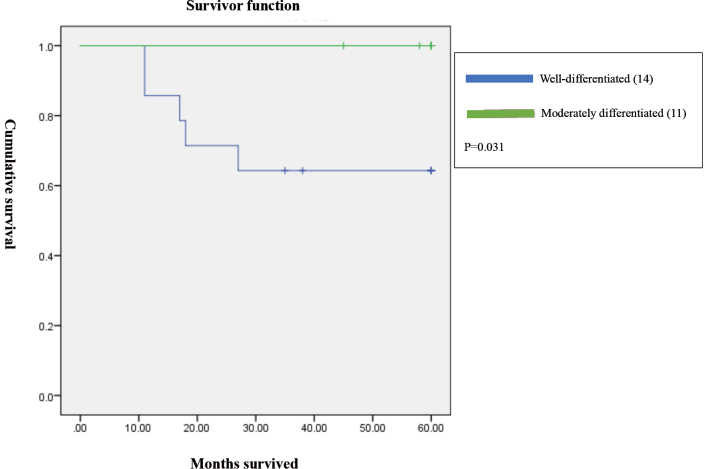
Survival curves based on extent of differentiation. There was a significant difference in survival rate between differentiation grades (p=0.031).

Patients with micrometastasis to lymph nodes had shorter survival compared to those without micrometastases (p=0.002; Figure 1). There was also a significant difference in survival based on the degree of differentiation (p=0.031; Figure 2). However, when pStage, micrometastasis, and degree of differentiation were input as independent variables in a Cox proportional hazard model, all three factors were not significant (pStage III or higher, hazard ratio (HR): 2.8, CI: 0.7-12, p=0.16; micrometastasis, HR: 2.7, CI: 0.8-9, p=0.11; well-differentiated, HR: 1.5, CI: 0.4-5.5, p=0.5). Based on the p-values, pStage III or higher and micrometastasis showed a tendency to be related to survival, but degree of differentiation did not do so (Table 4).

**Table 4:** Multivariate Cox regression analysis for degree of differentiation, Stage and micrometastasis

		Hazard ratio	Confidence intervals	p
Degree of differentiation	Well vs. Moderate	1.5	0.4 - 5.5	0.51
Stage	III, IV vs. I, II	2.8	0.7 - 12	0.16
Micrometastasis	Positive vs. Negative	2.7	0.8 - 9.1	0.11

## Discussion

The first finding in this study is that micrometastasis to lymph nodes is not related to the degree of differentiation in esophageal SCC. There are contradictory findings for the relationship of micrometastasis with the degree of differentiation in esophageal cancer. In our study, micrometastasis was associated with the degree of differentiation in univariate analysis and well-differentiated cancers were likely to have micrometastasis. However, this was not shown in multivariate analysis. We speculate that the results in univariate analysis may be due to bias since patients with well-differentiated tumors tended to have higher stage disease in this cohort.

Secondly, micrometastasis to lymph nodes was found to be a likely prognostic factor, even for locally advanced esophageal cancer. This might be a new finding because most previous studies have examined patients with early disease^3-6)^, but this finding is also consistent with previous results for various carcinomas, including esophageal carcinoma. On the other hand, the degree of differentiation was not a prognostic factor in multivariate analysis, which is inconsistent with previous studies showing that well-differentiated esophageal cancer is associated with good survival^7)^. This may be attributable to a difference of definitions between Japan and Western countries, since the degree of differentiation is based on the dominant type in Japan, but on the poorest type in Western countries. For example, when the well-differentiated region is dominant and the moderately differentiated part is small in esophageal cancer, the Japanese pathological diagnosis is well-differentiated, but the Western diagnosis would be moderately differentiated. Furthermore, our small cohort did not include any poorly differentiated cases, and this may also have affected the results.

There are several methods for diagnosis of micrometastases: immunostaining of lymph nodes, as performed in this study; lymph node detection by reverse transcription-polymerase chain reaction (RT-PCR); and immunostaining or RT-PCR using peripheral blood. One problem with use of resected lymph nodes is that the results are available only after surgery. Adjuvant therapy after esophageal cancer resection is not the standard in Japan, and there is no effective evidence-based adjuvant treatment, even if a poor prognosis is predicted. Recently, however, it has been shown that administration of nivolumab after esophagectomy following preoperative chemoradiotherapy leads to an improved prognosis in patients without pathological complete response (pCR)^12)^, and this treatment has been covered by health insurance in Japan from 2021. Micrometastasis-positive patients might be eligible for adjuvant therapy even if their carcinoma is pN0 or pN1.

This study has some limitations. First, it was not prospective and the number of patients was small. Second, in our small cohort, there were no cases of poorly differentiated esophageal cancer, and the analysis might have been affected by this biased population.

In conclusion, the results of this study suggest that micrometastasis to lymph nodes may be a prognostic factor, even in advanced esophageal cancer. The degree of differentiation was not related to micrometastasis or survival. Further studies are needed with more patients and addition of patients with poorly differentiated SCC to examine the underlying mechanisms.

## Funding

The authors received no financial support for the study.

## Author contributions

TAsakura performed data analysis and wrote the first draft of the manuscript.

HT corrected and approved the manuscript.

TM, AA contributed to material on pathology.

TAndo provided advice on micrometastasis and contributed to writing the manuscript.

NT, MT, YK made significant changes in revision of the manuscript.

All authors read and approved the final manuscript.

## Conflicts of interest statement

The authors declare that there are no conflicts of interest.
